# Marine Algae Polysaccharides: An Overview of Characterization Techniques for Structural and Molecular Elucidation

**DOI:** 10.3390/md23030105

**Published:** 2025-02-27

**Authors:** Kaitlin C. Lesco, S. Kim R. Williams, Lieve M. L. Laurens

**Affiliations:** 1 Laboratory for Advanced Separation Technologies, Department of Chemistry, Colorado School of Mines, Golden, CO 80401, USA; klesco@mines.edu (K.C.L.); skrwillia@mines.edu (S.K.R.W.); 2 Bioenergy Science and Technology Directorate, National Renewable Energy Laboratory, Golden, CO 80401, USA

**Keywords:** marine polysaccharides, characterization, separations, extractions

## Abstract

Polysaccharides make up a large portion of the organic material from and in marine organisms. However, their structural characterization is often overlooked due to their complexity. With many high-value applications and unique bioactivities resulting from the polysaccharides’ complex and heterogeneous structures, dedicated analytical efforts become important to achieve structural elucidation. Because algae represent the largest marine resource of polysaccharides, the majority of the discussion is focused on well-known algae-based hydrocolloid polymers. The native environment of marine polysaccharides presents challenges to many conventional analytical techniques necessitating novel methodologies. We aim to deliver a review of the current state of the art in polysaccharide characterization, focused on capabilities as well as limitations in the context of marine environments. This review covers the extraction and isolation of marine polysaccharides, in addition to characterizations from monosaccharides to secondary and tertiary structures, highlighting a suite of analytical techniques.

## 1. Introduction

Marine polysaccharides represent a diverse group of biopolymers derived from algae, bacteria, and other aquatic organisms, offering immense potential for sourcing bioactive compounds. Notably, these complex carbohydrates have garnered attention for their impressive functional properties, particularly their immunomodulatory effects, which can enhance immune responses, modulate inflammation, and serve as adjuvants in therapeutic formulations. The unique structural features of marine polysaccharides, such as sulfation patterns, unique monosaccharide compositions, branching patterns, interrelationships with other polymers, molecular weight, size, and conformation, are pivotal in dictating their rheological behavior, biological activity, and suitability for various pharmaceutical and commercial applications [1].

Compared to bioactive compounds sourced from terrestrial organisms, marine-derived biomaterials often exhibit higher levels of bioactivity and chemical diversity, making marine ecosystems a prolific source of structurally unique and biologically potent secondary metabolites [2]. This chemical innovation is driven by the extreme and varied conditions of marine environments, which have led to the evolution of distinctive biochemical adaptations in marine organisms. As a result, marine polysaccharides present a rich repository of bioactive compounds, offering novel scaffolds for drug discovery and development.

However, for the same reasons that marine polysaccharides have gained such significant interest, namely, their complex structures and unique chemical adaptations, they also present substantial challenges in their characterization and application. The intricate branching patterns, high molecular weight, and diverse monosaccharide compositions, often accompanied by unique modifications like sulfation or acetylation, complicate structural analysis [3]. Additionally, the high ionic strength and complex matrices from which these polysaccharides are extracted can interfere with conventional analytical techniques, impacting the reproducibility and reliability of results. These factors create significant hurdles in developing accurate structure-function models, essential for optimizing their therapeutic potential, particularly in drug delivery and immunomodulatory applications.

To ensure the successful integration of marine polysaccharides into pharmaceutical markets, stringent regulatory requirements must be met. Regulatory bodies like the FDA mandate detailed a characterization of these compounds, including their structural features, physicochemical properties, and biological activities [4]. Such comprehensive characterization is crucial not only for confirming product safety and efficacy but also for facilitating quality control and scalability in commercial production. Therefore, overcoming these analytical challenges is pivotal for advancing the use of marine polysaccharides in innovative drug formulations and harnessing their full potential as bioactive agents in marine-derived therapeutics.

**Figure 1:**
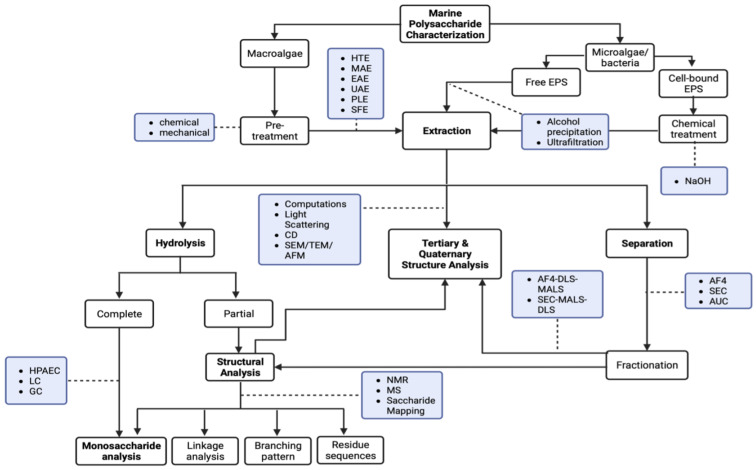
Workflow and techniques used to achieve different components of marine polysaccharide analysis. Extraction techniques include hydrothermal extraction (HTE), microwave-assisted extractions (MAE), enzyme-assisted extraction (EAE), ultrasound-assisted extraction (AUE), pressurized-liquid extraction (PLE), supercritical fluid extraction (SFE). Separation techniques can be conducted independently or prior to structural or monosaccharide analysis to form fractions using techniques such as asymmetrical flow field-flow fractionation (AF4), size exclusion chromatography (SEC), or analytical ultracentrifugation (AUC) paired with appropriate online detectors (such as multi-angle light scattering (MALS) and dynamic light scattering (DLS) for conformation determination, high-performance anion exchange chromatography (HPAEC), liquid chromatography (LC), and gas chromatography (GC) for monosaccharide characterization). Structural analyses use techniques such as nuclear magnetic resonance (NMR), mass spectrometry (MS) for saccharide mapping, computations, light scattering (LS), circular dichroism (CD), scanning electron microscopy (SEM), transmission electron microscopy (TEM), and atomic force microscopy (AFM) for determination of whole polymer analysis and supramolecular structures. Dotted lines indicate techniques which could be used for the respective analyses (arrows). Created using BioRender.com.

Conventional methods for polysaccharide analysis are hydrolysis of the polymeric forms, followed by chromatographic techniques, nuclear magnetic resonance (NMR), and mass spectrometry (MS) (as shown in Figure 1). Techniques like light scattering (LS), atomic force microscopy (AFM), and electron microscopy (EM) are necessary for examining higher-order structures and conformational properties. Although these techniques have proven effective for simpler polysaccharides like starch or cellulose, marine polysaccharides pose unique challenges. For instance, MS suffers from reduced ionization efficiency due to salts present in the native environment, while high ionic strengths and charged residues can reduce the effectiveness of size exclusion chromatography (SEC). Shear degradation of large charged MW polysaccharides can further affect SEC results. Analyzing marine polysaccharides and addressing these issues necessitate a shift in our analytical approach, especially when examining these complex, high-MW polysaccharides that lack the regular, repeatable unit structures found in semi-crystalline polysaccharides.

This review provides a critical assessment of the current landscape of analytical techniques for marine polysaccharide research, highlighting key characterization challenges and discussing how sample handling and preparation can influence the structural integrity of these biopolymers. It also emphasizes the need for methodological adaptations when analyzing marine polysaccharides, particularly in high-ionic-strength environments, to accurately reflect their native structures and bioactivities.

While previous reviews have made significant contributions to the field [5,6,7,8,9,10,11,12,13,14], many focus on specific aspects, such as extraction methods [15,16], characterization techniques for individual polysaccharides, or the application of a single analytical approach [17,18,19,20,21,22,23]. This review aims to address these limitations by offering a comprehensive evaluation of the full range of techniques employed across all structural levels of marine polysaccharides, from primary composition to higher-order conformation. It also identifies critical gaps in the current understanding of marine polysaccharide structures and bioactivities, particularly in the context of their immunomodulatory potential, and highlights areas where further research is needed. The structure–function relationship is an increasingly important topic because changes in a polysaccharide’s sulfate group, monosaccharide composition, MW, branching, glycosidic linkages, and conformation were found to correlate with the changes in its bioactivity and ultimately also with its hydrocolloid physical properties [24].

## 2. Overview of Marine Polysaccharides

Marine polysaccharides, in the context of this review, originate from macroalgae, microalgae, cyanobacteria, bacteria, and chordata [25]. In algae, polysaccharides can be found in the cell wall, intracellular space, and the extracellular environment as part of secreted extracellular polymeric substances (EPS). Both homopolysaccharides and heteropolysaccharides exist within the umbrella of marine polysaccharides either composed of one or multiple sugars, such as galactose (including anhydrogalactose), xylose, glucose, rhamnose, fucose, arabinose, and mannose, joined together with glycosidic linkages, and often decorated by various degrees of sulfation [6]. Commonly found polysaccharides include alginate, laminarin, fucoidan, agarose, porphyran, carrageenan, and ulvan in macroalgae [9,10] and cellulose, β-glucan, pectin, chitin, agaropectin, and lignin in microalgae and cyanobacteria [26]. The food industry commonly exploits some of these polysaccharides for their hydrocolloid thickening behaviors, such as the application of carrageenan, agar, and alginate in gels, gums, viscosity modifiers, and stabilizers [3,27]. Additionally, the recent literature demonstrates a growing interest in using marine polysaccharides as pharmacological ingredients based on their bioactivities, such as antibacterial agents, antitumor agents, and antioxidants [28,29,30,31,32]. Monosaccharide composition, branching and glycosidic linkages, MW, conformation, and the presence and degree of functional group substituents like sulfates all lead to different biological functions and properties of marine polysaccharides [10,33,34].

**Figure 2:**
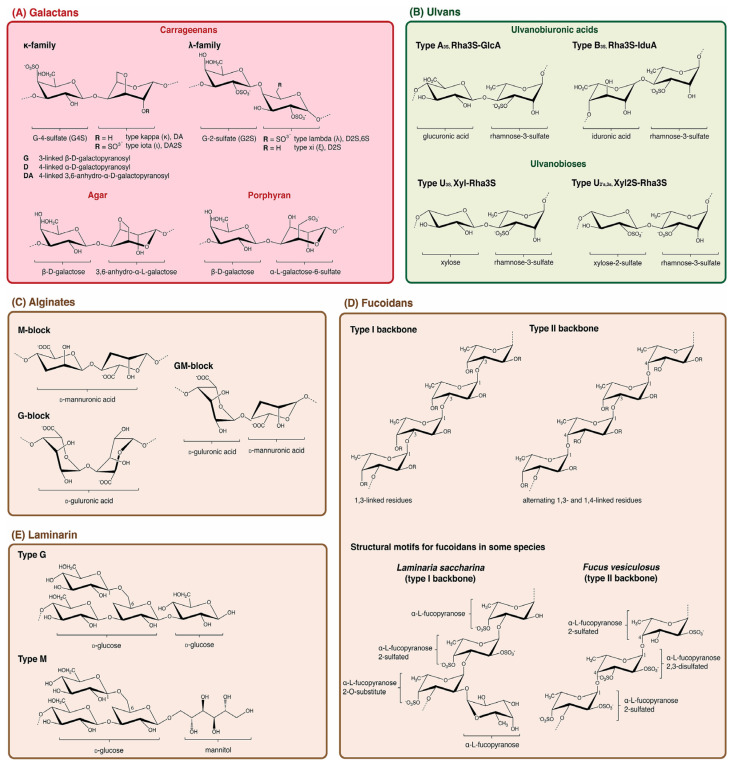
The major marine polysaccharides and their respective building blocks. Section (**A**) reports sulfated galactans, the major polysaccharide component in red seaweed. (**B**) reports ulvans, the major polysaccharide type found in green macroalgae. Sections (**C**–**E**) include polysaccharides primarily found in brown macroalgae. Figure reprinted from Sasaki and Yoshikuni, Metabolic engineering for valorization of macroalgae biomass. Metabolic Engineering (2022), 71, 42–61. Copyright 2022, with permission from Elsevier (license 5910390793984) [37].

The structure of polysaccharides is intimately related to their function and many marine polysaccharides have aleatory residues, making structural characterization difficult. For example, alginate is composed of two monomeric uronic acid residues: β-D-mannuronic acid (ManA) and α-L-guluronic acid (GulA) (Figure 2). A 1,4-linkage may randomly arrange this co-polymer into poly-ManA blocks, poly-GulA blocks, or ManA-GulA blocks [15]. Alginates with a higher GulA content have greater gelling properties, whereas alginates with a higher ManA content are linked to higher viscosities [15]. Additionally, carrageenans are polysaccharides yielding polymolecular and polydisperse mixtures of molecules. While carrageenan exists as a naturally occurring mixture of isomers, as shown in Figure 2 (κ-, ι-, and λ-carrageenans all of which exhibit varying degrees and locations of sulfation in the 3,6 anhydrogalactose monomers), the extraction process can induce changes in the distribution of the respective isomers. Alkaline extractions can convert λ-carrageenan to κ- or ι-types, convoluting our certainty in the native structure, as well as affecting the properties that lead to end-use applications [3]. The degree of sulfation has been shown to affect bioactivities. For example, higher sulfation led to an increase in the anti-HIV activity of κ-carrageenan [35]. Similar to alginate, the ratio of isomers will impact the overall structure and properties of the biopolymer and can either induce or impede gelling [36].

**Figure 3:**
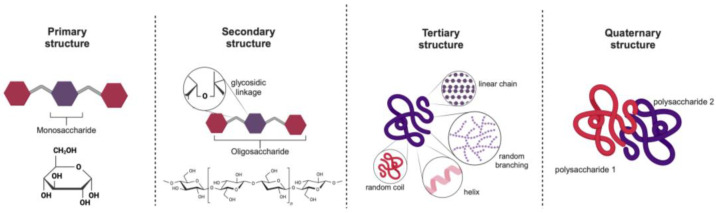
Figure representing the described four structural levels of polysaccharides. Created using BioRender.com.

Polysaccharides exhibit a hierarchical structural organization, similar to that found in proteins, as depicted in Figure 3 [17]. At the primary level, they consist of monosaccharide building blocks. The secondary level considers the patterns of the glycosidic linkages along the polysaccharide chain. The tertiary structure refers to the overall conformation, which may include various shapes such as linear, helical, rod-like, random coil, worm-like, or spherical forms. Finally, the quaternary structure describes the supramolecular arrangements, including aggregation phenomena [17,38]. Each structural level influences the distinct properties and functional behavior of polysaccharides, impacting their suitability for various applications. For instance, ions present in marine environments can lead to ion-mediated conformational shifts, which can lead to gelation [39].

## 3. Preparation of Polysaccharides: Extraction and Purification

Sample preparation poses an important first step in the determination of the structure and conformation of the polysaccharide, as the method of extraction and hydrolysis can have unintended effects [40,41]. Here, we discuss some of the common methods and their respective impacts on the quality of the isolated polymer. While this section will be brief, the effects that extraction can render on a polysaccharide structure necessitates its inclusion in this review.

### 3.1. Extraction Techniques for Polysaccharides Internal to the Organism

Extraction techniques vary largely depending on the originating organism and the type of polysaccharide. An efficient extraction for process development must be high-yielding and of high purity, environmentally sustainable, economical, and potentially suitable for commercial application. For a bench-scale characterization of polysaccharides, these extraction qualities may not all be necessary. However, one must still be aware of the structural changes that may be incurred through the extraction procedure. Extractions typically use some form of chemical or mechanical method followed by centrifugation or filtration to isolate the polysaccharides from the algal cells. One potential challenge when attempting to extract polysaccharides from marine organisms using low-severity extraction techniques, such as enzyme-assisted extraction in their natural high ionic strength environment, is a lower extraction efficiency due to a reduction in osmotic pressure and, subsequently, a reduction in cell wall disruption [42,43]. However, when comparing polysaccharides extracted from a freshwater environment to those from a saltwater environment, similar bioactivities were observed. Therefore, if obtaining a high extraction yield is a desired outcome, it may be beneficial to extract marine polysaccharides from algae in a low ionic strength environment or adopt an extraction method focused on a physical separation.

#### 3.1.1. Macroalgae Applications

Removing internal polysaccharides is a much more challenging task compared to extracellular ones, especially for those in macroalgae due to the recalcitrant nature of the structural surface or barrier polymers. This leads to the need for pretreatment. Pre-treatment has two major purposes: (1) to isolate the polysaccharides via removing other bound macromolecules and impurities and (2) to break down the cell wall [16]. Solvent-based pre-treatments to remove lipids and pigments have been performed on the biomass using acetone [44,45], acetone supplemented with ethanol [44], or methanol [46]. Furthermore, mechanical pretreatment to break down the cell wall can be conducted using milling [47,48], washing [49], and sonication [50]. Conventional extractions after pre-treatment include solvent or hydrothermal extraction (HTE) with water or a dilute acid or base performed at high temperatures for extended periods [51]. Since these conventional extraction techniques are thought to be either harsh on the environment, not sustainable, scaleable, and not economical due to the energy demands, new technologies have emerged, such as microwave-assisted extraction (MAE), pressurized liquid extraction (PLE) [52], ultrasound-assisted extraction (UAE), enzyme-assisted extraction (EAE) [53], sub-critical water extraction (SWE), or thermal treatment sequentially after conventional extraction (specifically HTE), to increase extraction efficiencies [54]. Choosing an extraction technique will depend on the type of species or biomass, the desired polysaccharide specificity of the extraction, and the end-use application of the polysaccharide.

It is understood that extraction techniques can impact yield and alter the functionality of different fucoidans. Alboofetileh et al. recently investigated the effects of different extraction methods on the antibacterial and antiviral properties of fucoidans from *Nizamuddinia zanardinii* (Phaeophyceae) [55]. Sub-critical water extraction was demonstrated to be the highest-yielding technique compared to MAE, UAE, EAE, HTE, and hybrid methods, with the lowest yield coming from UAE. The differences in yield are attributed to differences in the technique’s ability to dismantle the cell wall and release the polysaccharides, allowing them to diffuse into the extraction solvent. The SWE extraction combines high temperatures and pressures, which effectively allow for improved solvent penetration and cell destruction. Methods such as SWE can increase yields but may also induce depolymerization or desulfation. It was reported that the fucoidans extracted via SWE had the lowest sulfate content and the lowest number average molecular weight only after enzyme–ultrasound-assisted extraction. More interesting was the change in bioactivities observed using different extraction methods. All methods yielded fucoidans with anti-viral activity against HSV-2. The antiviral activity is controlled by the anionic nature of the sulfate, which inhibits viral adsorption. The antibacterial activity, which is controlled by the monosaccharides, sulfate, and phenolic content, was more selective for the extracted polysaccharides. Fucoidans extracted using MAE and SWE inhibited *E. coli*, and fucoidans extracted using hybrid techniques like enzyme–ultrasound, ultrasound–microwave, and SWE inhibited *P. aeruginosa* [55]. In addition to having a relatively low molecular weight and the lowest degree of sulfation, the fucoidans extracted using SWE had the greatest abundance of uronic acids which all contributed to the broad antimicrobial activity observed.

In addition to fucoidans, the depolymerization of carrageenans has been demonstrated in some cases to favor bioactivities, such as the anti-tumor properties, which are higher at lower molecular weights [56]. Molecular weight, however, is not the only property that can undergo changes during extraction, as studies have observed transitions from λ-carrageenan into κ- or ι-carrageenans under alkaline extraction conditions. This transition leads to a reduction in the degree of sulfation, resulting in conformational changes and, consequently, changes associated with the carrageenans’ propensity to form gels [3,56]. Jönsson et al. previously published a comprehensive review of extraction techniques for brown, red, and green algae, comparing techniques and yields for different polysaccharides from different species of algae [9].

#### 3.1.2. Microalgae and Bacteria Applications

The extraction procedures for internal polysaccharides in microalgae and bacteria are like those of macroalgae, however, often less intensive. As such, the conventional method of extraction is often utilized without the need for pre-treatment. Traditional hot-water extraction for microalgae is similar to protocols for macroalgae that often consist of elevated temperatures and slightly acidic or basic environments for some period of time, followed by precipitation with a solvent, typically ethanol [6,32,57,58,59]. Facile optimizations of traditional hot-water extraction protocols by modifying pH, extraction temperature, and duration and subsequent ultrasonication have been shown to efficiently extract high yields of polysaccharides from microalga *Tribonema minus* (Xanthophyceae) [57]. Similar experiments optimizing hot-water extraction using response surface methodology have been demonstrated on *Rhodosorus* sp. (Rhodophyta) SCSIO-45730 [32] and have proven to be an important tool in extraction and process optimization.

There are various methods used to extract microalgae when hot-water extraction is not effective in breaking down cell walls and dissolving polysaccharides or increasing extraction yield. Some commonly used extraction methods include alkali, ultrasound, and enzyme-assisted methods, similar to those used in macroalgae [60]. Additionally, methods such as supercritical carbon dioxide (CO_2_) extractions have been successfully applied to the extraction of polysaccharides from microalgae. Moreover, it was found that the polysaccharide extracts from *Auxenochlorella pyrenoidosa* (formerly *Chlorella pyrenoidosa*) (Chlorophyta) had higher antiradical activities compared to traditional solvent exactions making supercritical CO_2_ extractions a promising non-toxic alternative aimed at specific applications in the antioxidant and nutraceutical industry [61]. The above reinforces the idea that an extraction method should be chosen based on the type of algae, type of polysaccharide or crude, and commercial application, and all extraction methods should include an exploration of critical parameters.

### 3.2. Extraction Techniques for Polysaccharides External to the Organism

Extraction techniques for polymers external to the organism are aimed at isolating the polysaccharides or extracellular polymeric substances secreted by microalgae and bacteria from their surrounding environment. Recently, it was demonstrated that macroalgae also exude dissolved organic carbon molecules, many of which are (sulfated) polysaccharides. Specifically, *Fucus vesiculosus* (Phaeophyceae) demonstrated secretion of 0.3% of their biomass in fucoidan per day [62]. While exuded polysaccharides have been documented in the literature from macroalgae sources, they are far less abundant than those from microalgae and bacteria. For this reason, the extraction techniques will be focused on external polysaccharides of microbial origin. Due to their natural presence outside the cell, extraction and isolation become much less intensive compared to internal polysaccharides. The primary challenges are instead associated with a sometimes low abundance of polymers of interest, requiring the processing of large volumes. Some microalgae are known to secrete large quantities of extracellular polymeric substances, with some reaching up to 71% of their biomass weight [63].

The extraction of extracellular polysaccharides requires only isolation from a cell-free supernatant. As such, the extraction process requires an initial separation of algal/bacterial cells from the media. This is usually achieved by centrifugation followed by vacuum filtration of the supernatant to remove any additional cells or impurities [64]. The polysaccharides are then isolated from the supernatant by either (1) precipitation with alcohol, [58,65] usually ethanol, or (2) ultrafiltration or dialysis [64,66,67]. Afterward, crude polysaccharides are obtained by either freeze-drying or oven-drying. Of the two extraction techniques, alcohol precipitation is the most selective for polysaccharides. However, in high-saline environments, salts will co-precipitate [8]. Filtration-based extraction methods may be more appropriate at high ionic strength, but they are not selective for polysaccharides and will isolate the entire EPS matrix, including bound proteins and other impurities in the supernatant.

For cell-bound EPSs, the extraction procedure requires additional steps. If the EPS is tightly bound, it requires more rigorous methods, such as high temperatures and chemical extraction, while loosely bound EPSs may have their extraction assisted with low temperatures or centrifugation [68]. Takahashi et al. have recommended the most efficient method of extraction of bound EPSs from benthic diatoms when compared to other physical and chemical methods, which is cation exchange resin extraction [69]. This technique involves using a resin with charged functional groups that exchange cations (e.g., Na^+^ or H^+^) with the ionic polymers in the EPS, thereby desorbing the EPS from the diatom surface after separation from the water matrix. In contrast, studies looking at bound EPSs from the cyanobacterium *Microcystis wesenbergii* found that chemically-assisted NaOH extraction was the most appropriate method in terms of efficiency when comparing five other chemical and physical extraction methods [70].

### 3.3. Purification Methods

After extraction, purification of the crude polysaccharide may be desired. In some cases, purification is necessary to increase the accessibility and digestibility of polysaccharides when they are intertwined in matrices of proteins and lipids, such as in the case of EPS [71]. The objective of purification could be to separate the polysaccharides by their type or to remove compounds that were co-extracted with the polysaccharides or desalt the polysaccharides. For desalting, simple procedures such as dialysis or performing a buffer exchange in SEC can be facilitated. For the isolation of specific types of polysaccharides, a common technique uses an affinity chromatography column such as a DAED (Diethylaminoethyl–Sepharose) column to purify based on ion interactions [72]. Both ion exchange chromatography and size exclusion chromatography will be covered in the separation section (Section 4) in more depth.

## 4. Separation Techniques for Molecular Weight Distribution Analysis

**Figure 4:**
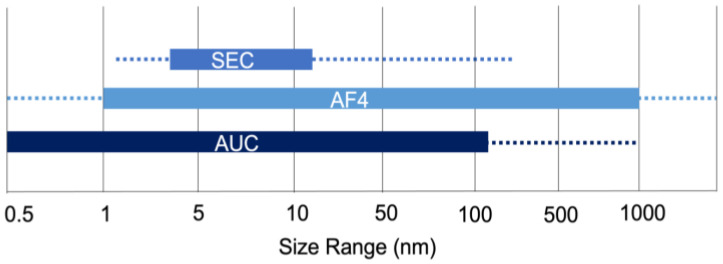
Ideal size ranges for different size separation techniques, including size exclusion chromatography (SEC), asymmetrical flow field-flow fractionation (AF4), and analytical ultracentrifugation (AUC), demonstrated in solid blocks with dashed lines demonstrating instrument capability range.

Molecular weight (MW) is one of the more important characteristics of polysaccharides as related to many physical and mechanical properties and bioactivities of the polysaccharide [1]. Many natural marine polysaccharides are polydisperse and can have ultrahigh molecular weights contributing to their complex primary structures [22]. This makes a separation technique with MW determination capabilities an important step in characterization. Additionally, a successful separation method can be applied to enrich fractions for later analyses, which can reduce detection interferences and the complexity of the sample. A good separation for MW determination of polysaccharides should have the following characteristics: resolve different MW populations, when possible, cover a large dynamic range, provide good sample recovery, limit sample adsorption, and limit the shearing effects that bias MW. All of the following techniques will focus on the separation of macromolecules, with a special emphasis on the use of online detectors. The application of more than one detector can provide an independent and orthogonal measurement of molecular weights of the different size populations as they elute from the separation column or channel. While all the following techniques are capable, considerations regarding the separation scale (e.g., preparative or analytical), molecular weight range, and ionic nature of the carrier fluid and sample should be taken into account when choosing the most appropriate method. The following sections aim to discuss the limitations and advantages of each technique, while the ideal polymer ranges are featured in Figure 4.

### 4.1. Size Exclusion Chromatography (SEC)

Size exclusion chromatography can provide estimates of the molecular weights for analytes, in addition to separating and preparing fractions and desalting samples. In order to separate analytes by size, size exclusion chromatography employs a rigid (or gel, in the case of gel permeation chromatography) porous packing material as the stationary phase and a liquid mobile phase to separate molecules by size [73]. Various packing materials can be used depending on the mobile phase and analyte compatibility, as well as the analytes’ size range.

While often paired with online detectors capable of determining size or MW such as light scattering techniques, calibration plots established with polymer or globular protein MW standards are commonly used, which allow for extrapolation of the analyte MW. The latter provides a quick, cost-effective method for determining the MW of proteins but problems arise in applying such a calibration plot to polysaccharides, since the conformation of polysaccharides rarely is globular or known. Regardless, MWs for polysaccharides have been estimated using calibration plots with pullulan standards for polysaccharides from *G. caudata* [74]. Online multi-angle light scattering (MALS) coupled with differential refractive index (dRI) detectors can be used to obtain absolute MW data, as well as conformation and size [75].

Another consideration affecting retention and analyte interactions with the packing material when using SEC is the mobile phase. For marine polysaccharides, some analyses can be conducted in water (or water with dilute sodium azide to deter microbial growth) [76], while other polysaccharides are insoluble in water [73]. In one study, it was demonstrated that chromatogram characteristics, including the shape and retention time repeatability, improved with the use of sodium nitrate as a carrier fluid rather than water [77]. When analyzing marine polysaccharides in their native high ionic strength environment, a reduction in intramolecular electrostatic interactions may occur, shifting the retention time of the analytes [78]. Difficulties may arise in distinguishing between retention time shifts from changes in the electrostatic interactions of polymer and packing material versus polymer–polymer aggregation, so utilizing an online detector capable of absolute MW determination becomes increasingly important for an accurate reporting of molecular properties when the mobile phase is not optimized.

One challenge when analyzing ultrahigh molecular weight polysaccharides is the potential of shear degradation occurring, even if the pore sizes nominally permit separation of that molecular weight range [79,80]. It has been demonstrated that polystyrene standards with MWs over 10^7^ g/mol undergo shear degradation in an SEC column [81,82]. For marine polysaccharides, shearing can bias the results, leading to a systematic underestimation of molecular weight. Additionally, while one may choose to extrapolate analyte MW using calibration plots, the lack of good (mono-disperse, conformation known) polysaccharide standards with the same hydrodynamic volume as the sample may lead to erroneous results. Regardless of shearing and nonideal interactions, SEC remains the most-used size-based separation method for marine polysaccharides.

SEC has been extensively used to investigate the MWs of marine polysaccharides. The MWs of fucoidan, alginic acid, and laminarin were estimated using SEC with dextran standards [83]. In order to extend the MW range of the separation, two ultrahydrogel columns (2000 and 500) were coupled. The MWs were estimated to be 250, 200, and 5–64 kDa, for fucoidan, alginic acid, and laminarin, respectively. Similarly, the MW of ulvan polysaccharides from stranded *Ulva* sp. has also been determined using SEC. Based on the extraction approach, different MWs were obtained between 201 and 1841 kDa [84].

### 4.2. Asymmetrical Flow Field-Flow Fractionation (AF4)

An alternative to packed-bed chromatography is asymmetrical flow field-flow fraction (AF4), where the absence of a stationary phase allows for an extended range of MW polymers that can be separated and analyzed [85]. In normal mode AF4, the separation is based on the analytes’ diffusion coefficients, whereby smaller analytes elute first followed by large, resulting in a size-based separation [85,86,87,88]. The separation of analytes occurs in an open ribbon-like channel that lays upon a permeable membrane surface, making the technique well suited for ultrahigh MW analytes, as the degree of shearing is reduced significantly compared to SEC [89]. Therefore, AF4 has a wide dynamic range, as pictured in Figure 4, and is only limited on the low end by the molecular weight cut off of the membrane.

Considerations for the separation of marine polysaccharides include the membrane chemistry, molecular weight cut-off, and carrier fluid used. Two of the most commonly used membrane types for AF4 analyses are regenerated cellulose (RC) and polyethersulfone (PES). While the separation is diffusion-based, the adsorption of analytes onto the membrane may occur, albeit to a lesser extent than SEC, because of the lower surface area of the FFF channel [90]. Compared to SEC, the absence of packing material also allows for the use of a wide variety of carrier fluids, which may enable analyses in the analyte’s native environment. For analytes with limited solubility like polysaccharides, high ionic strength analyses not only give more accurate characterizations of polysaccharides and their molecular properties in their native environment, leading to structure–function elucidation, but also provide a workaround to precipitation caused by insolubility.

Although the application of AF4 for marine polysaccharides is largely unexplored, promising results have been obtained in studies conducted on standards of polysaccharides produced by marine microorganisms [89]. More recently, publications using AF4-MALS-dRI to investigate bacterial EPS have demonstrated the successful application of AF4 for marine polysaccharides [91,92].

κ-Carrageenan has been investigated using AF4 coupled to multiangle light scattering (MALS) detection to determine the molecular weight and conformation [93]. The conformational properties were examined with and without anionic surfactant sodium stearoyl lactylate. It was determined that the MW without surfactant present was 3 × 10^5^ Da. However, in the presence of surfactant, the MW increased to 12 × 10^5^ Da. The conformation was also estimated using root mean square radius (*R_rms_*) and MW to calculate the Flory exponent, indicating a more compact conformation of κ-carrageenan polysaccharide in the presence of sodium stearoyl lactylate.

Kang et al. studied the persistent length of chitosan polysaccharides at different ionic strengths using AF4, demonstrating that AF4 separates chitosan with less adsorption than SEC at ionic strengths below 100 mM [94]. Furthermore, they explored the physical properties of chitosan in an acetate buffer at a range of ionic strengths from 1.25 to 800 mM, observing a decrease in persistent length with an increasing ionic strength [94].

Infernan, an extracellular polymer secreted by *Alteromonas infernus*, has been studied using AF4 to evaluate its molecular properties following chemical modifications aimed at enhancing its anticoagulant and anticancer activity [92]. Those two chemically altered samples were analyzed. One was subjected to radical depolymerization and the other to additional sulfation. The MWs of these modified polysaccharides were determined to be 27 kDa and 57 kDa, respectively.

### 4.3. Analytical Ultracentrifugation (AUC)

Analytical ultracentrifugation (AUC) experiments use high-speed centrifugal forces to provide data on MW, macromolecular interactions, and the non-ideal behavior of macromolecules in solutions, typically aqueous buffers, viscous matrices like sucrose or glycerol gradients, or other polar solvents [95,96]. The technique separates analytes based on their sedimentation and diffusion behavior under centrifugal force, enabling the detection of analytes across a wide size range, from 10^3^ to 10^9^ Daltons [97]. Detection in AUC is typically achieved using optical systems, such as absorbance detectors for UV–visible wavelengths, interference optics to measure changes in refractive index, or fluorescence detectors for enhanced sensitivity with labeled analytes. The choice of detector depends on the analyte’s properties and the experimental goals, making AUC highly versatile for analyzing proteins, polysaccharides, and synthetic polymers in both simple and complex media [96].

AUC provides several distinct advantages, including its non-destructive nature, solution–phase analysis, and ability to analyze multiple samples simultaneously [97]. However, certain challenges persist, such as the inability to easily collect fractions of separated components in real-time and the extended duration of experiments, which can span from several hours to days [98]. Additionally, differentiating analytes with similar physical properties can be difficult without the integration of complementary detection techniques [97]. Non-ideal behaviors, including analyte–analyte interactions, often complicate sedimentation analysis. This is particularly relevant for charged biopolymers like marine polysaccharides, which may aggregate or exhibit other concentration-dependent phenomena [99,100].

Due to these complexities, particularly in determining the molecular weight solely using AUC for polysaccharides, hybrid techniques have been explored. For instance, coupling SEC with AUC, where SEC first separates components by size before AUC measures molecular weight through sedimentation equilibrium, has proven effective [101]. Despite AUC’s challenges, comparative studies, such as those analyzing chitosan with both SEC-MALS and AUC, have demonstrated comparable molecular weight results between these methods [102]. Furthermore, research on alginates from *Laminaria hyperborea* (Phaeophyceae) has highlighted the impact of thermodynamic non-idealities like exclusion volumes and Donnan effects under low ionic strength conditions. These studies revealed that insufficient charge suppression at low ionic strengths could lead to underestimated molecular weights at lower sample concentrations [103].

## 5. Techniques for the Determination of Primary Structures (Monosaccharides)

At the basis of any conceptual chemical and physical model of polysaccharides is the monomeric composition and organization. The monosaccharide content is considered an important factor of the polysaccharide structure, as it will largely impact the immunomodulating properties of the polysaccharide, with examples such as fucose associated with anti-inflammatory and anticoagulant properties [104] and mannose associated with immunostimulatory activities [105]. Additionally, enhancing the bioactivities of the polysaccharides through modifications such as sulfation relies on the specific composition and availability of their monosaccharide building blocks [106]. Monitoring and quantifying these constituent monosaccharides depend highly on the optimization of the chromatography and detection methods used, as well as the effectiveness of hydrolysis prior to analysis. The methodology for monosaccharide analyses has been well-established and can be accomplished using several different techniques. It often takes the form of a full (acid or enzyme) hydrolysis followed by monomer separation and detection. We have highlighted below the hydrolysis methods followed by the most common techniques for constituent monosaccharide identification. Thanks to the complete hydrolysis, one can forgo the difficulties presented by the high MW and heterogeneity of marine polysaccharides while investigating the monosaccharides.

### 5.1. Hydrolysis Methods

An important step in preparing polysaccharides for primary structure and secondary structure (oligosaccharide and branching level) analyses is hydrolysis. Acid hydrolysis is the most common thanks to its effectiveness, hydrolysis rate, and cost-effective nature. However, other techniques (such as enzymatic hydrolysis) exist and may be beneficial for linkage analysis and structural preservation during partial hydrolysis or when acid hydrolysis is too aggressive and produces secondary degradation products, as discussed below.

#### 5.1.1. Complete Hydrolysis

Complete hydrolysis is used to prepare the polysaccharides for primary structure analysis by breaking down the samples to their monosaccharide sugars. For complete hydrolysis, acids are most often used and include trifluoroacetic acid [34,41,107], sulfuric acid [108], and hydrochloric acid [109]. Hydrolysis has also been performed using bases like KOH [110]. The optimization of acid concentration, time, and temperature needs to be considered to avoid hydrolyzing beyond completion. Without optimizations, destructive hydrolysis can occur, leading to the acid-catalyzed dehydration of glucose to hydroxymethylfurfural (HMF) [111]. If interested in monitoring glucose isomers and whether monosaccharides are pyranose or of the straight chain form, the type of acid used in the hydrolysis will need to be considered. Previous experiments demonstrated that, with HCl hydrolysis, nearly 60 mol% of straight-chain glucose was created compared to the 32 mol% generated using sulfuric acid [112].

#### 5.1.2. Partial Hydrolysis

Partial hydrolysis is an important aspect of oligosaccharide structure determination, which allows for probing the branching patterns and molecular organization. The type of hydrolysis used here can vary and has varying results on the remaining structure. Techniques include dilute acid hydrolysis and enzymatic hydrolysis. Different experimental variables contribute to the degree of hydrolysis and have also been shown to affect oligosaccharide yield [113]. In the recent literature, the effects of weak-acid thermal hydrolysis conditions on oligosaccharide yield were studied. It was determined that, while temperature and acid concentration affected oligosaccharide yield, the time of hydrolysis was the most significant factor affecting the yield of oligosaccharides from *Eucheuma denticulatum* (Rhodophyta) seaweed [113]. An ideal temperature range of 120–130 °C and an acid range of 0.9–1.6 M (sulfuric acid) is considered optimal for partial hydrolysis, where parameters outside of these ranges negatively affect yields.

Another common (often partial) hydrolysis method is enzymatic hydrolysis. Different types of enzymes can be used for different types of cleavages, making enzymatic hydrolysis highly selective. For example, selective cleavage can be conducted by using endo- or exo-enzymes, whereby endo-enzymes would provide random cleavage of the glycosidic linkages, and exo-enzymes commonly cleave monosaccharide or oligosaccharide fragments sequentially from the non-reducing end [114]. Geun Goo et al. (2013) demonstrated the use of α-amylase and α-glucosidase to hydrolyze EPS into glucose [71]. Enzymatic hydrolysis is considered non-destructive and does not pose a risk of acid-catalyzed dehydration of monosaccharides, making it appropriate for partial or complete hydrolysis.

Hydrogen peroxide has also been used for chemical depolymerization of polysaccharides to oligosaccharides. Comparing the results between hydrochloric acid hydrolysis and enzymatic hydrolysis, hydrogen peroxide revealed structural differences between the respective oligosaccharides, resulting in superior antioxidant activities for the hydrolysates prepared with hydrogen peroxide [115]. These findings demonstrate that different methods of hydrolysis result in different structures with varying degrees of polymerization, sulfate content, and reduced sugar content, which ultimately affect the antioxidant potential of algal polysaccharides [115].

### 5.2. Chromatographic Separation and Detection of Monosaccharides

Once the constituent monosaccharides are prepared using hydrolysis, a suitable technique must be used for the separation, detection, and quantification of the sugars. The monosaccharides most often found in algae include glucose, xylose, rhamnose, galactose, fucose, arabinose, mannose, myo-inositol, ribose, glucosamine, galactosamine, uronic acids such as glucuronic acid, galacturonic acid, and mannuronic acid, and deoxygenated sugars and sugar derivatives [116]. Chromatography is used as the technique of choice for the separation and selective detection of monosaccharides. Understanding the monosaccharide composition is the first step to identifying the compositional differences that lead to highly distinct physical properties among complex higher-ordered polymer structures.

#### 5.2.1. High-Performance Anion Exchange Chromatography (HPAEC)

High-performance anion exchange chromatography (HPAEC) relies on the ionization of saccharides in high-pH environments and allows for the determination of constituent monosaccharides and oligosaccharides in polysaccharide samples [117]. It is considered the highest-resolution method for the separation of monosaccharides among different natural polysaccharides, due to its ability to differentiate sugars based on their dissociation constants [118]. As the working principle is based on the ionization ability of monosaccharides, even isomeric sugars can be discriminated [119].

The most commonly used detector for HPAEC is pulsed amperometric detection (PAD), which enables picomole-range detection limits without requiring prior derivatization due to specialized waveforms optimized for sugars, with the added benefit that the waveform can be optimized for the simultaneous sensitive detection of both neutral sugars, deoxy-sugars (e.g., fucose and rhamnose), amino-sugars (e.g., glucosamine and galactosamine), and uronic acids [120,121].

A thorough evaluation of multiple chromatography methods, including HPLC (using Prevail and Shodex Pb^2+^ columns), GC with alditol acetate derivatives, and HPAEC-PAD, indicated that HPAEC is the highest-resolution technique to separate the 13 monosaccharides found in microalgal carbohydrates [116]. Their findings revealed that only HPAEC-PAD could distinctly separate all sugars without introducing any extraneous peaks from by-products.

Generally, PAD can tolerate variations in salt concentration, allowing for analysis in various ionic strength and pH environments. However, the retention times of sugars can be significantly influenced and need to be considered when building and validating HPAEC-PAD methods [121,122]. Often, desalination prior to analysis is needed, which complicates the direct analysis of marine samples. However, the structural information gained from monosaccharides should not be affected by the analysis environment.

HPAEC-PAD analysis has also been employed for the investigation of oligosaccharides. The enzymatic hydrolysis of fucoidans from *Fucus vesiculosus* using *Lentimonas* sp. CC4 has been examined using HPAEC-PAD to monitor the degradation products [123]. The results suggest that extracellular exo-enzymes are responsible for the digestion of fucoidans. Additional examples of the application of HPAEC for the detection and separation of marine polysaccharides are summarized in Table 1.

#### 5.2.2. Gas Chromatography (GC)

Gas chromatography (GC) is a widely used technique for analyzing volatile derivatized monosaccharides derived from polysaccharide samples and can be particularly useful for the determination of linkages in complex polymers [124]. The derivatization step enhances volatility, protects against and minimizes thermal degradation, and can improve detector sensitivity. Derivatization methods often involve silylating agents to produce trimethylsilyl (TMS) derivatives or acetylation to form alditol acetates [46,112]. While alditol acetate derivatization generates a single chromatographic peak per monosaccharide, it is unsuitable for uronic acids (due to a lack of a reducing carbonyl group), which are better analyzed using TMS derivatives, albeit at the cost of producing multiple peaks [64,125,126]. GC is compatible with various detectors, such as flame ionization detectors (FID), thermal conductivity detectors (TCD), and mass spectrometry (MS), offering flexibility for different analytical applications [127].

GC is valued for its speed and cost-effectiveness compared to techniques like HPAEC or HPLC. It also provides a superior resolution of monosaccharides in certain contexts, as evidenced by studies showing better performance for GC in resolving free sugars compared to HPLC [116,128]. However, GC requires the desalting of marine samples to maintain optimal peak shape and resolution, and derivatization can be labor-intensive. Additionally, some derivatives may degrade during analysis, and the chromatograms can become complex due to the formation of multiple isomeric peaks, except in the case of alditol acetate derivatives [122].

GC analysis has been widely employed for the analysis of marine polysaccharides including the concurrent detection of sugars and metabolites [129] and amino-sugars from algal organic matter [130]. Additional examples of GC analysis used for the analysis of marine monosaccharides are presented in Table 1.

#### 5.2.3. High-Performance Liquid Chromatography (HPLC)

High-performance liquid chromatography (HPLC) is a versatile technique for the separation, identification, and quantification of monosaccharides. HPLC uses a liquid mobile phase, allowing for the partitioning of analytes based on column stationary phase interactions, driven by solubility, size, charge, or polarity [131] with one or more detectors, commonly a diode array detector (DAD), UV-vis detector, mass spectrometer, and differential refractive index (dRI). Often the separation of similar saccharides is challenging and relies on the respective selective separation capacity of the stationary phase of the column selected. For example, for aqueous mobile phases, normal or adsorption chromatography are used [131], with reverse-phase liquid chromatography a common choice for saccharide separation, which is reliant on derivatization of the polar monomers to allow for high-resolution separation on non-polar stationary phases, such as C18 or C8, and a polar water–organic solvent mobile phase [132]. Reverse-phase liquid chromatography enables shorter retention times for nonpolar analytes and is particularly effective at minimizing interference from salts, allowing for the analysis of samples in high-ionic strength environments [122]. Another common form of HPLC is hydrophilic interaction liquid chromatography (HILIC), which employs the use of a polar stationary phase with organic solvents [133].

While HPLC offers the benefit of accommodating multiple online detectors, it generally exhibits lower resolution than GC or HPAEC, often resulting in poor separation of certain sugars and multiple chromatographic peaks for others [122]. Depending on the detector used, derivatization may also be necessary to attach a chromophore to the monosaccharide to increase the method’s sensitivity to low-concentration analytes or increase the resolution of similar anomers [117]. Despite its limitations, HPLC has been commonly used to analyze marine polysaccharides due to its widespread availability in lab spaces.

HPLC has proven effective in specialized applications. For example, studies have employed zwitterionic HILIC columns paired with high-resolution MS to monitor ^13^C-labeled monosaccharides from EPS, providing insights into polysaccharide turnover [67]. Other works have used PMP (1-phenyl-3-methyl-5-pyrazolone) derivatization to optimize separation and detection on C18 columns with MS detection [134]. Furthermore, HPLC has been applied to study secondary structural features, offering valuable data beyond compositional analysis, which will be covered in Section 6.

Additional examples of HPLC applications for the detection and analysis of marine polysaccharides are summarized in Table 1.

**Table 1:** Different chromatographic techniques for the separation of mono- and oligosaccharides.

Polysaccharide Source	Species	Technique	Detector	Derivatization	Structural Information	References
Extracellular polymeric substances	*Porphyridium* *Sordidum* (Rhodophyta)	HPLC ^1^	MS ^2^, UV ^3^	PMP ^4^	Primary	[107]
	*Porphyridium purpureum* (Rhodophyta)	HPLC	MS, UV	PMP	Primary	[107]
	*Chaetoceros socialis* (Mediophyceae)	HPAEC ^5^	PAD ^6^	None	Primary	[135]
Fucoidans and Fucans	*Sargassum vulgare* (Phaeophyceae)	HPLC	dRI ^7^	None	Primary	[45]
	*Fucus serratus* (Phaeophyceae)	HPAEC	PAD	None	Primary, Secondary	[123]
	*Cladosiphon okamuranus* (Phaeophyceae)	HPAEC	PAD	None	Primary, Secondary	[123]
	*Fucus vesiculosus* (Phaeophyceae)	HPAEC	PAD	None	Primary, Secondary	[123]
	*Durvillaea potatorum* (Phaeophyceae)	HPAEC	PAD	None	Primary, Secondary	[123]
	*Ecklonia maxima* (Phaeophyceae)	HPAEC	PAD	None	Primary, Secondary	[123]
	*Halopteris scoparia* (Phaeophyceae)	GC ^8^, HPAEC	MS, PAD	TMS ^9^ (for GC)	Primary	[136]
	*Cystoseira compressa* (Phaeophyceae)	GC	MS	TMS	Primary	[46]
Alginate	*Halopteris scoparia* (Phaeophyceae)	GC, HPAEC	MS, PAD	TMS (for GC)	Primary	[136]
	*Saccharina japonica* (Phaeophyceae)	HPLC	dRI	None	Primary	[137]
	*Cystoseira compressa* (Phaeophyceae)	GC	MS	TMS	Primary	[46]
Laminarin	*Laminaria digitata* (Phaeophyceae)	HPAEC	PAD	None	Primary	[138]
	*Saccharina latissimi* (Phaeophyceae)	HPAEC	PAD	None	Primary	[138]
	*Sargassum henslowianum* (Phaeophyceae)	GC	MS	PMAA ^10^	Primary, Secondary	[139]
Ulvan	*Ulva fasciata* (Chlorophyta)	GC	MS	TMS	Primary	[140]
	*Ulva lactuca* (Chlorophyta)	HPAEC	PAD	None	Primary	[141]
	*Ulva rigida* (Chlorophyta)	HPAEC	PAD	None	Primary	[142]
Carrageenan	Unspecified	HPLC	MS	PMP	Primary, Secondary	[143]
	*Kappaphycus alvarezii* (Rhodophyta)	GC	FID ^11^	Alditol acetate	Primary	[144]
	*Chondrus crispus* (Rhodophyta)	HPLC	MS	None	Primary, Secondary	[145]
Unspecified	*Gracilaria gracilis* (Rhodophyta)	HPAEC	PAD	None	Primary	[146]
	*Nannochloropsis* sp. (Eustigmatophyceae)	HPAEC	PAD	None	Primary	[108]
	*Emiliania huxleyi*	GC	MS	TMS	Primary	[129]

^1^ High-performance liquid chromatography; ^2^ mass spectrometry; ^3^ ultraviolet visible spectroscopy; ^4^ 3-methyl-1-phenyl-2-pyrazolin-5-one; ^5^ high-performance anion exchange chromatography; ^6^ pulsed amperometric detection; ^7^ differential refractive index; ^8^ gas chromatography; ^9^ trimethylsilyl derivatives; ^10^ partially methylated alditol acetate; ^11^ flame ionization detector.

## 6. Techniques for the Determination of Secondary Structures (Oligosaccharides)

The analysis of marine polysaccharides’ secondary structure becomes increasingly difficult, as the analysis must determine the sequence of sugar residues along with their linkages. However, in addition to MW and monosaccharide composition, the glycosidation branching will determine the therapeutic properties of the polysaccharide. More specifically, the anti-oxidant and anti-coagulant capacities of fucoidan have been linked to branching, yet the branching patterns of the majority of fucoidan polysaccharides have yet to be elucidated [147]. Furthermore, laminarin extracted from different species exhibits varying degrees of β-1,3 and β-1,6 linkages, resulting in unique structural properties and bioactive characteristics [148]. Performing these analyses in a high ionic strength marine environment is increasingly challenging and is compounded by additional difficulties arising from instrument limitations. Determining the secondary structure of polysaccharides requires the use of multiple analytical techniques. Commonly used techniques include nuclear magnetic resonance (NMR), mass spectrometry (MS), matrix-assisted laser desorption ionization mass spectrometry (MALDI-MS), and various chromatographic techniques. After a partial hydrolysis of the oligosaccharides, these techniques can provide insight into the substitution or branching pattern of oligosaccharide fragments of polysaccharides.

The important research field of glycomics, which represents the study of glycan structure–function relationships, provides a valuable approach to the characterization of secondary structures of polysaccharide complexity. Furthermore, glycomics can often expand beyond the study of polysaccharides to describe the interactions of carbohydrates with other macromolecules, such as lipids (glycolipids) and proteins (glycoproteins) [149]. As the forefront of glycomics relies on understanding the structure–function relationship, the study can be particularly useful in elucidating the mechanisms behind the immunomodulating properties that make marine polysaccharides of interest. Already a large body of glycomics literature exists, with some examples including investigations of the antiparasitic activity of sulfated polysaccharides and the use of additives to increase the production of carbohydrates in microalgae [150,151].

Understanding the secondary structure of polysaccharides is crucial to comprehending the tertiary and quaternary structures and further supports the conceptual building of a polysaccharide model. It also helps in investigating the mechanisms that guide the rheological characteristics of the polysaccharide, e.g., the linkage patterns that promote polysaccharide gelling in certain environments [152]. Note that the following section is not inclusive of all techniques used for characterization but rather covers the most employed techniques and techniques that provide the most information that can be translated into structure elucidation. Techniques such as FTIR and XRD are also commonly used in characterization, but since the information provided by these techniques only represents bulk properties, we have omitted including them in this review. For more detail regarding the application of these techniques for polysaccharide characterization, the reader is referred to a recent review published by [153].

### 6.1. Nuclear Magnetic Resonance (NMR)

Nuclear magnetic resonance (NMR) can provide a suite of information regarding molecular composition, conformation, and interactions, making it an indispensable tool for structural elucidation and dynamic studies of marine polysaccharides [154,155]. Moreover, NMR has the added advantage of allowing for native environment insights without damaging the sample.

NMR experiments can vary depending on the nuclei being monitored and/or the state of the analyte (solution or solid). Additionally, experiments can generate one chemical shift axis (1D NMR) or two (2D NMR) to deduce the complexity of the spectra if needed. Most monitored isotopes for polysaccharides are ^1^H and ^13^C [155]. Both techniques allow for discrimination of the anomeric proton or carbon using 1D NMR. Certain chemical shifts can indicate whether the α- or β-configuration of an anomeric proton or carbon is present. In proton NMR, chemical shifts at *δ* 5.34–5.37 with ^3^J_1,2_ coupling constants valued below 4.0 Hz helped Cai et al. identify the presence of α-D-glucose in Limnospira platensis (formerly *Spirulina platensis*) (Cyanobacteria) oligosaccharides [41]. Meanwhile, Cheong et al. (2022) identified chemical shifts of 4.6 ppm and 102.8 ppm for the anomeric proton and carbon, respectively, in D-glucopyranosyl while analyzing *Laminaria digitata* (Phaeophyceae) oligosaccharides. These chemical shifts were indicative of the β-anomeric conformation of laminarin and distinguished this glucan polymer from cellulose or starch glucans [156].

For more complex cases of structural determination, 2D-NMR or correlation spectroscopy can be used to provide information on individual sugar residues and sequences of sugar residues (long-range homo- or heteronuclear correlation) [155]. Hao et al. (2019) utilized 1D ^1^H and ^13^C NMR along with 2D 1H-13C heteronuclear single quantum coherence (HSQC), 1H-1H correlation spectroscopy (COSY), and heteronuclear multiple bond correlation (HMBC) spectra for the investigation of a novel polysaccharide from *Caulerpa chemnitzia* (formerly *Caulerpa racemosa* var. *peltate*) (Chlorophyta). The approach facilitated the determination of the linkage sequence of seven sugar residues, culminating in a proposed detailed structure [157]. Infernan from *Alteromonas infernus* (bacteria) was analyzed for its structural composition using proton NMR, along with HSQC, COSY, 1H-1H total correlation spectroscopy (TOCSY), HMBC, and rotating-frame Overhause effect spectroscopy (ROESY) correlation spectroscopies [158]. Despite employing several NMR techniques, a complete structural analysis was performed by combining ESI-MS and partially methylated alditol acetate-derivatize GC analysis with NMR, demonstrating the importance of a multi-faceted approach for structural elucidation of complex polysaccharide structures.

Regarding the analysis of marine polysaccharides, the effectiveness of NMR can diminish due to limitations with high MW heterogeneous samples, resulting in a loss of sensitivity (and peak broadening) due to low tumbling rates and shorter relaxation times. Additionally, the spectra become increasingly complicated with large macromolecules due to more NMR-active nuclei [159]. Hydrolysis can address high MW concerns, but it may lead to complexity in the spectrum from diverse monosaccharides and coinciding chemical shifts [160]. Additional challenges may arise if trying to perform an analysis in the native high-salinity environment, especially with the use of cryoprobes. Cryoprobes rely on superconducting circuits and operate at extremely low temperatures to enhance sensitivity. The high ionic strength of saline in marine samples increases conductivity, leading to excessive dielectric heating and electrical noise, which can interfere with the probe’s performance and compromise its delicate electronics. This sensitivity to ionic strength results in decreased experimental sensitivity [161]. Developments in the design of NMR tubes regarding the shape and size of the sample cavity and magnetic susceptibility of the material have helped circumvent the sensitivity losses that accompany NMR analyses at high ionic strengths [162]. Voehler et al. recorded NMR measurements at ionic strengths of 4 M (6–7 times the ionic strength of seawater) by altering the geometry of the NMR tube without severely compromising the signal-to-noise ratios. While these tubes may not represent what is commercially available, Varian and Bruker have developed salt-tolerant cold NMR probes [163].

Beyond its limitations, NMR analysis has been frequently utilized for the successful analysis of marine polysaccharides, including the elucidation of substituents, as demonstrated by Dore et al. (2013) who identified sulfated and glycosylated carbons through chemical shifts between 105 and 74 ppm. Additionally, NMR analyses have been used independently to ascribe α-carrageenan spectra [164], as well as elucidate α-glucan [165] and β-glucan structures [166]. Advancements in data analysis tools such as CASPER have made computation-assisted chemical shift prediction and spectral evaluation available, facilitating a more nuanced and accurate interpretation of the spectra from complex marine polysaccharides [167,168]. The structure of the polysaccharide-based bioflocculants from *Streptomyces* sp. (bacteria) [169] have been investigated using CASPER for automated structural elucidation.

### 6.2. Mass Spectrometry (MS)/Matrix-Assisted Laser Desorption Ionization (MALDI)

Mass spectrometry is a powerful technique that can be used for the molecular elucidation of marine polysaccharides and provides information on absolute or relative molecular quantitation depending on the instrument and the sample [170]. With the use of ultrahigh-resolution MS instrumentation, it is possible to determine the elemental composition through highly accurate mass-to-charge ratios, while fragmentation can help elucidate the structural composition and configurations. While powerful for the identification of unknowns, mass spectrometry is also challenging in the context of quantification of respective contributions of the oligosaccharides identified because, more often than not, standards for the short-chain oligosaccharides identified are not commercially available. Additionally, challenges exist in the identification of the oligomers and glycans because of the lack of publicly available database resources for the automated analysis and processing of glycomics data.

Mass spectrometry can be a highly sensitive analytical method that provides more molecular resolution and identification than NMR. It offers fast analysis times and can provide unambiguous structure assignment through fragmentation [171]. MS can be used independently (e.g., direct infusion analysis with electrospray ionization, ESI) or as an online detector following a separation method, which reduces the complexity of polysaccharide mixtures. When analyzing complex and heterogeneous samples, mass spectrometry, like NMR, is not immune to challenges, e.g., the loss of mass accuracy and resolution as the MW of the sample increases or ion suppression when the complexity of the samples overwhelms the detectors. To increase the identification and quantification of high MW polymers, hydrolysis can be applied, partial or complete, and reduce the size distribution. Like NMR, the heterogeneity of hydrolysates may result in multiple peaks, some of which overlap, leading to overly complex spectra.

There is a challenge with marine salts that are non-volatile and can negatively affect mass accuracy, signal, detection limits, and resolution through the distribution of analyte peaks over multiple salt adducts [172,173]. Furthermore, the presence of salts can increase chemical noise and cause ion suppression [173]. These effects not only harm the spectral quality but also cause non-volatile salts to precipitate in the MS source, leading to equipment wear over time. Reduction of the ESI emitter tips to sub-micron sizes has been demonstrated to reduce the salt cluster ions. However, this requires non-conventional instrument modifications [174].

Several examples of the characterization of complex marine oligosaccharides have been demonstrated using MS techniques. For instance, carrageenan and agarose from red macroalgae have been characterized using ESI-CID (collision-induced dissociation)-MS/MS on Q-TOF instruments [175,176]. Similarly, ESI-CID-MS/MS has been used in the structural determination of alginates from *L. japonica* [177]. Furthermore, ESI-Orbitrap-MS, in addition to MALDI-MS (tandem TOF, TOF), have been used to investigate sulfated polysaccharides and anionic porphyran [178,179]. Yang et al. (2009) investigated galactan polysaccharides, including κ-carrageenan and agarose, in which detailed sequences were determined by ESI-MS in the negative mode, and the results were corroborated using CID to create informative fragments. While analyzing agarose, the molecular weights were investigated in positive-ion mode. Then, the experiments were switched to negative-ion mode for sequence analysis [176].

An easier way to mitigate limitations caused by high molecular weight and the presence of salts is to use MALDI-MS. MALDI-MS is commonly used for marine polysaccharide characterization. MALDI is a soft ionization surface sampling technique that is reliant on secondary ionization from an applied matrix and allows for the analysis of analytes [180,181,182].

Commonly used matrices for native and derivatized polysaccharides are 2,5-dihydroxybenzoic acid (DHB) and α-Cyano-4-hydroxycinnamic acid (CHCA) for glycopeptides [183]. Liquid matrices like glycerol are also used, which provide good stability and signal-to-noise ratios [184], or ionic liquid matrices like HABA/TMG_2_ in the context of glycomics profiling of complex hetero-polysaccharides [179]. While MALDI-MS has been demonstrated to have a higher salt tolerance compared to traditional MS techniques, investigations into matrices such as 3, 4-diaminobenzophenone (DABP) for improved salt tolerance have been conducted [185]. However, traditional matrices of DHB and CHCA perform similarly, demonstrating minimal ion suppression in the presence of non-volatile salts [185,186]. Derivatization before analyzing samples with MALDI-MS is not required. However, it can be conducted to increase the mass interval between oligomers, which makes unambiguous assignments easier. Chizhov et al. demonstrated this by analyzing methylated derivatives of laminarin polysaccharides [187]. This increased the mass interval between G-chain and M-chain oligomers from 2 mass units to 16 mass units, making them more distinguishable, especially at high degree of polymerization (d.p.) values.

In addition to high salt tolerances, another benefit of using MALDI-MS over conventional (infusion-based) MS is that it produces mostly singly charged ions with minimal fragmentation. This is because MALDI is a soft ionization technique. As a result, it is a suitable method for characterizing the mass of complex polysaccharide mixtures [183]. Depending on the mass analyzer, MALDI can also have a large mass-profiling range, making the analysis suitable for both higher MW polymers and low MW oligosaccharides [188]. One reported challenge with MALDI-MS for polysaccharide analysis is low ion yields. Studies have demonstrated increased ion yields through *a priori* derivatizations [189]. However, matrix application alone can make sample preparation for MALDI-MS analysis time-consuming. Additional steps, such as derivatizations, further contribute to laborious sample preparations.

Other experiments set out to use NMR as a technique for structural elucidation of the sulfated polysaccharides from *Capsosiphon fulvescens* (Chlorophyta). Several homonuclear and heteronuclear correlation techniques (COSY, ROESY, HSQC, and HMBC) were applied but yielded no evidence of hexoses. As such, two mass spectrometry techniques, ESI-MS and MALDI-MS, were utilized to complete the investigation. The positive-ion mode identified a series of alternating saccharide units, even though sulfates can be labile in this mode. Major peaks in negative-ion mode were also identified, which supported the presence of alternating saccharide units [178]. While this study highlights the limitations of NMR techniques, such as the requirement for high sample concentrations compared to MS, its results more strongly emphasize the imperative need to employ multiple analytical techniques in tandem to elucidate complex structures of marine polysaccharides [177,178].

### 6.3. Saccharide Mapping and Molecular Probes

Saccharide mapping is a suite of techniques that use partial hydrolysis followed by a chromatographic separation for the determination of the polysaccharide structure [190,191]. While only a few studies exist on saccharide mapping techniques, and many techniques vary from the type of hydrolysis (acid or enzyme) to the chromatographic technique (HPLC, TLC, SEC, and carbohydrate gel electrophoresis (PACE)) [190,191,192,193], examples of saccharide mapping for marine polysaccharide structural determination are available. S. Chen et al. (2021) used saccharide mapping for the characterization of brown seaweed polysaccharides. They utilized specific enzymolysis followed by derivatization with PMP for HPLC-UV analysis to separate and observe hydrolysates and test for positive reactions. Positive reactions to enzyme-catalyzed hydrolysis indicate different structural information depending on the enzyme used. Chen et al. were able to detect the presence of β-D-glucans and β-1,3-1,4 type polysaccharides from positive responses to cellulase and lichenase. Positive responses to α-amylase and pectinase indicated the presence of α-D-glycosidic linkages. Lastly, a positive response to α-fucosidase indicated that fucoidan-type saccharides were present [194]. While saccharide mapping alone is not capable of full structural elucidation, it has been proven to be a highly informative complementary technique.

To further analyze using carbohydrate-active enzymes, carbohydrate-binding modules (CBM) can be incorporated. Carbohydrate-binding modules are non-catalytic domains that help enzymes bind to carbohydrates, enhancing their efficiency. CBMs with affinity to alginate have been used to facilitate the degradation of the polysaccharide [195]. In addition to binding with polysaccharides, CBMs can exist independently as probes. The probes act as affinity attachments that bind to single polysaccharide epitopes, allowing for the localization of specific carbohydrate motifs [196]. In addition to CBMs, monoclonal antibodies (mAbs) have been used as molecular probes in the research of algae polysaccharides. Diatom fucan polysaccharides were investigated using both CBMs and mAbs to detect temporal fluxes in polysaccharide structures [135]. The bound probes, through the use of a secondary antibody, exhibit a colorimetric response, which allows for a calculation of the polysaccharide concentration. This analytical approach allows for the study of polysaccharide fluctuations, lending insights into the stability of the polysaccharide over time.

## 7. Macromolecule and Supramolecular Organization Determination

Analyzing the tertiary and quaternary structures of polysaccharides presents another difficult challenge, mainly due to the requirement in many cases to have a priori knowledge of their secondary structure and the limitations of available techniques. The conformational flexibility of polysaccharides contributes to the difficulties in elucidating their structures. Studying these substances in their native environment is crucial to gaining insight into their indigenous structures. Variations in ionic strength and the presence of ions significantly influence their conformational dynamics and supramolecular arrangements, including aggregation. Additionally, careful consideration of the techniques used to extract polysaccharides is necessary to ensure that their macromolecular structure is not affected. For instance, some techniques can change the degree of sulfation, which in turn, can alter the conformation of the polysaccharide. Higher degrees of sulfation encourage a rigid rod structure, while a reduction in the sulfate substituents results in a random coil conformation [3,92].

### 7.1. Light Scattering and Circular Dichroism

Light scattering (LS) is a non-destructive method that enables real-time observation of the molecular characteristics of marine polysaccharides, often used as an online detector to differentiate between various conformations in polydisperse samples. Unfortunately, LS analyses may experience difficulties associated with differentiating individual polysaccharides from supramolecular aggregates, and interpretation of results will suffer with increased polydispersity of the polysaccharides. While LS itself is not sensitive to ionic strength, the conformation and aggregation of polysaccharides are heavily influenced by the ionic strength of the environment [197]. Nevertheless, if LS is being used as an online technique, one must also consider the adverse effects of high salt concentrations on the separation. For example, branching patterns of fucoidans from *Fucus vesiculosus* (phaeophyceae) were studied using batch LS techniques, in which the root mean square radius (R_rms_) was determined from the initial slope of Berry dependences (MALS) and the hydrodynamic radius (R_h_) from DLS. The ratio of R_rms_ to R_h_ was used calculate the form factor (ρ), where ρ = R_rms_/R_h_, with different values indicating distinct conformaitons. The results demonstrated form factor values of ρ = 0.8–1.3, suggesting a symmetrical shape that is dependent on the ionic strength of the solution that the fucoidans were suspended in [197]. Similarly, an analysis of sulfated polysaccharides from the red alga *G. blodgettii* indicated a ρ value of 1.57 indicating a linear polysaccharide with a random coil conformation [198].

A complementary spectroscopic technique to study conformations of molecules is circular dichroism (CD) which uses the difference between absorbances of right or left polarized light [199]. Differential absorbances occur in chiral molecules and are measured as a function of wavelength to generate a CD spectrum, whereas polysaccharide structures are determined using multidimensional potential energy surfaces [200]. J. Liu et al. (2020) used CD to investigate the conformational changes in *Sargassum fusiforme* fucoidans under different extraction methods. Helix-like and sheet-like structures were indicated through observed cotton effects at 191 and 209 nm. Additionally, the observed cotton effect for HCl extractions indicated a change in conformation as a result of different extraction methods, stressing the impact of extractions on polysaccharide conformations [201].

### 7.2. Computational Simulation

Computational chemistry offers an ideal approach to probe the different effects of salts and ionic strengths without the limitations of conventional instrumentation. However, some idealities that may occur in experimental conditions may not be accounted for when exclusively relying on computations, challenging the routine and broad application. Unlike other macromolecules such as proteins, homology modeling cannot be applied to carbohydrates due to their inherent flexibility and the resultant lack of one singular three-dimensional structure [202]. There are two main classes of force fields for polysaccharide modeling; the first of which assumes all interactions are from van der Waals interactions, with the exception of the dihedral (φ) angle, known as the hard sphere exo-anomeric force field (HSEA). The second guiding philosophy assumes that the sum of bond stretching and bending, torsional rotations, and non-bonding interactions is equal to the potential energy. Force fields that follow the second philosophy include AMBER, CHARMm, and GROMOS. For smaller oligosaccharides, the MM2 and MM3 forcefields are commonly used [202].

However, one significant obstacle to using computational methods is the necessity of possessing a comprehensive understanding of the secondary structure of polysaccharides, rendering computational analysis quite challenging for newly discovered polysaccharides, including those found in EPSs. For example, the conformational structures of the EPS Infernan from *Alteromonas infernus* were recently solved using a multistep approach involving molecular mechanics (MM), molecular dynamics (MD), and density functional theory (DFT) [203]. First, MM was used to identify the lowest energy conformation. Second, MD was used to determine the most populated state over time, investigate the formation of helices, and probe the Ca^2+^ binding site. Finally, DFT was used to further investigate the Ca^2+^ binding sites. They discovered that the lowest energy conformation changed in the presence of calcium ions from a 2-fold helix to a 5-fold helix. They also proposed a mechanism for gel formation in which rotation from a 5-fold to a 2-fold helix induces chain pairing [203].

## 8. Conclusions

This literature review explores the complexities of characterizing marine polysaccharides and the techniques used in their analysis. It also discusses the challenges posed by the high ionic strength environment and the inherent complexity of these macromolecules. The review highlights the impact of extraction or hydrolysis processes on the overall macromolecule and its biological activities. While marine polysaccharides have emerged as attractive biomaterials with high-value bioactivities, the existing analytical methodologies, which are primarily designed for comprehensive structural elucidation, still require further development to provide complementary insights into their complex properties.

Despite significant progress in understanding the structural aspects of isolated marine polysaccharides, numerous questions persist regarding their intricate structures and dynamic interactions in vivo. The extent to which these polysaccharides intertwine, along with the intricacies of their interactions with other macromolecules and ions in both the marine environment and within organisms, remains enigmatic. Structural characterization faces challenges in the presence of micro-heterogeneities and is exacerbated in the case of major heterogeneities within complex polysaccharides making up the structural integrity of the originating organisms or plants. Additionally, the environmental conditions during polysaccharide analysis influence their structural conformation, yet limited research exists on studying marine polysaccharides in their native environment due to the challenges associated with a high-salt environment. Consequently, uncertainties remain across all structural levels of marine polysaccharides. These complex unanswered questions underscore our aim to decode the enigmas of marine polysaccharides in vivo starting with outlining the current state-of-the-art characterization techniques.

Looking ahead, dedicated efforts to advance macromolecular characterization in high ionic strength environments hold the promise of deeper insights into marine polysaccharides and, in turn, will pave the way for more effective utilization in various high-value applications.

## Data Availability

No primary data was generated as part of this work.
